# Mesenchymal stromal cells as prophylaxis for graft-versus-host disease in haplo-identical hematopoietic stem cell transplantation recipients with severe aplastic anemia?—a systematic review and meta-analysis

**DOI:** 10.1186/s13287-021-02170-7

**Published:** 2021-02-04

**Authors:** Ruonan Li, Jingke Tu, Jingyu Zhao, Hong Pan, Liwei Fang, Jun Shi

**Affiliations:** Regenerative Medicine Clinic, National Clinical Research Center for Blood Diseases, State Key Laboratory of Experimental Hematology, Institute of Hematology and Blood Diseases Hospital, Chinese Academy of Medical Sciences & Peking Union Medical College, No. 288 Nanjing Road, Heping District, Tianjin, 300020 China

**Keywords:** Mesenchymal stromal cells, Severe aplastic anemia, Hematopoietic stem cell transplantation, Haplo-identical, Graft-versus-host disease, Meta-analysis

## Abstract

**Background:**

Mesenchymal stromal cells (MSCs) are an emerging prophylaxis option for graft-versus-host disease (GVHD) in haplo-identical hematopoietic stem cell transplantation (haplo-HSCT) recipients with severe aplastic anemia (SAA), but studies have reported inconsistent results. This systematic review and meta-analysis evaluates the efficacy of MSCs as prophylaxis for GVHD in SAA patients with haplo-HSCT.

**Methods:**

Studies were retrieved from PubMed, EMBASE, Cochrane, Web of Science, and http://clinicaltrials.gov from establishment to February 2020. Twenty-nine single-arm studies (*n* = 1456) were included, in which eight (*n* = 241) studies combined with MSCs and eleven (*n* = 1215) reports without MSCs in haplo-HSCT for SAA patients. The primary outcomes were the incidences of GVHD. Other outcomes included 2-year overall survival (OS) and the incidence of cytomegalovirus (CMV) infection. Odds ratios (ORs) were calculated to compare the results pooled through random or fixed effects models.

**Results:**

Between MSCs and no MSCs groups, no significant differences were found in the pooled incidences of acute GVHD (56.0%, 95% CI 48.6–63.5% vs. 47.2%, 95% CI 29.0–65.4%; OR 1.43, 95% CI 0.91–2.25; *p* = 0.123), grade II–IV acute GVHD (29.8%, 95% CI 24.1–35.5% vs. 30.6%, 95% CI 26.6–34.6%; OR 0.97, 95% CI 0.70–1.32; *p* = 0.889), and chronic GVHD (25.4%, 95% CI 19.8–31.0% vs. 30.0%, 95% CI 23.3–36.6%; OR 0.79, 95% CI 0.56–1.11; *p* = 0.187). Furtherly, there was no obvious difference in 2-year OS (OR 0.98, 95% CI 0.60–1.61; *p* = 1.000) and incidence of CMV infection (OR 0.61, 95% CI 0.40–1.92; *p* = 0.018).

**Conclusions:**

Our meta-analysis indicates that the prophylactic use of MSC co-transplantation is not an effective option for SAA patients undergoing haplo-HSCT. Hence, the general co-transplantation of MSCs for SAA haplo-HSCT recipients may lack evidence-based practice.

## Introduction

Severe aplastic anemia (SAA) is a life-threatening bone marrow failure syndrome characterized by pancytopenia and hypoplastic bone marrow. Hematopoietic stem cell transplantation (HSCT) has been considered as a first-line therapy for young adults [1]. However, only 20–30% acquired SAA patients realistically hope to find a human leukocyte antigen (HLA)-matched sibling donor. With the improvement of conditioning regimens, like “Beijing protocol,” haplo-identical HSCT (haplo-HSCT) has recently been widely used to treat SAA patients as an alternative strategy [2]. However, the main challenges facing current haplo-HSCT usage included the risk of graft-versus-host disease (GVHD) and a higher graft failure (GF) rate [3–5]. Therefore, improving the haplo-HSCT outcomes in SAA patients is of great concern.

Mesenchymal stromal cells (MSCs) are multipotent stromal cells characterized by modulating immune and inflammation response, supporting hematopoiesis, and repairing tissues, which are widely used in haplo-HSCT [6, 7]. MSCs can be isolated from many tissues, including bone marrow (BM), cord blood and umbilical cord (UC) tissues, periosteum, adipose tissue, and fetal liver [8, 9]. According to several previous clinical studies, the application of MSCs in haplo-HSCT can decrease the incidence and severity of acute or chronic GVHD, promote facilitation of HSC engraftment, and improve OS [10–15]. However, others found that MSCs may make little or no difference in reducing the risk of GVHD and death [16–18]. Thus, these conflicting results need to be addressed urgently [19].

To the best of our knowledge, there have been some excellent clinical studies about proposing haplo-HSCT as the first-line therapy for SAA patients [20–22]. Therefore, it is of great importance to clarify key factors related to the outcomes of SAA with haplo-HSCT. For example, some meta-analyses compared different donor sources in haplo-HSCT, evaluating whether peripheral blood (PB) or BM as graft source produces a more satisfactory outcome in SAA patients [23], while others sought the optimal conditioning regimen for haplo-HSCT in patients with SAA [24–26]. In addition, several meta-analyses have approved the efficacy of MSCs in haplo-HSCT recipients with hematological conditions, mostly in hematological malignancies [27, 28]. Nevertheless, no meta-analysis has been done to evaluate the efficacy of MSCs combined with haplo-HSCT in SAA patients so far. Therefore, we performed the first systematic review and meta-analysis to investigate the efficacy of MSC co-transplantation following HSCT in patients with SAA.

## Methods

### Literature search

This meta-analysis was performed according to the Preferred Reporting Items for Systematic Reviews and Meta-Analyses (PRISMA) guidelines issued in 2009 [29]. We performed a systematic literature search in PubMed, EMBASE, OVID, Web of Science, and Cochrane Central Register of Controlled Trials (CENTRAL) from inception to January 2020 with the search terms “haplo-identical hematopoietic stem cell transplantation,” “mesenchymal stem cells,” and “severe aplastic anemia.” In addition, we searched clinical trials in http://clinicaltrials.gov with “severe aplastic anemia [condition/disease] AND (haplo-identical hematopoietic stem cell transplantation [other terms]).”

### Study selection

Articles in the literature were identified and data were extracted by two investigators independently. Disagreements were resolved through discussion. The reference lists of relevant studies were also hand-searched. These searches were limited to the “first generation” reference lists. We removed duplicates and reviewed titles or abstracts. Studies that met the following criteria were included: (1) phase 2 or 3 clinical trials or retrospective studies evaluating the efficacy of MSC co-transplantation following HSCT in patients with SAA, (2) cases with > 5 patients, (3) studies with consistent criteria of observation items, and (4) studies reported a quantitative outcome of interest. Exclusion criteria were the following: (1) review papers or expert opinions, (2) individual case reports, (3) studies did not report a quantitative outcome of interest, (and 4) studies were reported in a language other than English. Meta-analyses do not involve human subjects and do not require Institutional Review Board review.

### Data extraction

Data extraction from the eligible studies was carried out independently by 2 authors. We used a standardized extraction form to extract information about the first author, year of publication, study design, number of patients, median age, median intervals from diagnosis to treatment, the prophylaxis of GVHD, the conditioning regimen, acute GVHD (aGVHD), grade II–IV aGVHD, chronic GVHD (cGVHD), engraftment rate, all-cause mortality rate, and cytomegalovirus (CMV) infection rate. Because there are differences in study design, we extracted information in accord with the following criteria: (1) if data were used in two or more studies, data from the latter study were extracted on the basis of subtracting data published in the formal study; (2) we also summarized hematologic reconstitution time without performing a meta-analysis.

### Quality assessment

Two authors worked independently on quality assessment. If disagreements occurred, an adjudicator was consulted. Single-arm studies were assessed using the Newcastle-Ottawa Scale modified for cohort studies without controls, as previously used by Lopez-Olivo [30]. Potential scores ranged from 0 to 6, with higher scores indicating higher quality. The following components were assessed: selection, which includes the representativeness of the exposed cohort; ascertainment of exposure; demonstration that the outcome of interest was not present at the start of the study; and outcome, which consists of an assessment of outcome, followed up long enough for outcomes to occur, and adequacy of the follow-up of cohorts. Because it was a meta-analysis of single proportions, publication bias was not advisable in this study.

### Statistical analysis

Data manipulation and statistical analyses were performed using Stata statistical software version 15.0 (StataCorp, College Station, TX, USA) and R software (version 3.6.3). We conducted separate analyses for single-arm retrospective researches studying the treatment outcomes of haplo-HSCT with MSCs or without MSCs. Heterogeneity between studies was evaluated via *I*-squared statistic and *p* value. When heterogeneity was significant (*p* < 0.05 or *I*-squared > 50%), a random-effects model was adopted to pool the results. Then, *x*^2^ tests were applied to find if there are statistical differences of pooled estimates between groups, the effect measure was the adjusted odds ratio (OR) with 95% confidence intervals (CIs), and *p* < 0.05 was defined as statistically significant. The results of the meta-analysis were graphically displayed by forest plots and heterogeneity was further explored by subgroup analysis and sensitivity analysis. Notably, the event rates can be zero or one from some studies yet they still need to be included in the analysis to represent the whole population. In such cases, the resulting distribution of proportions tends to be 0 inflated. We made good use of the Freeman-Tukey double arcsine transformation to perform normalization and variance, which can be achieved by using the metaprop module to perform fixed and random effects meta-analysis of proportions.

## Results

### Search results

**Fig. 1:**
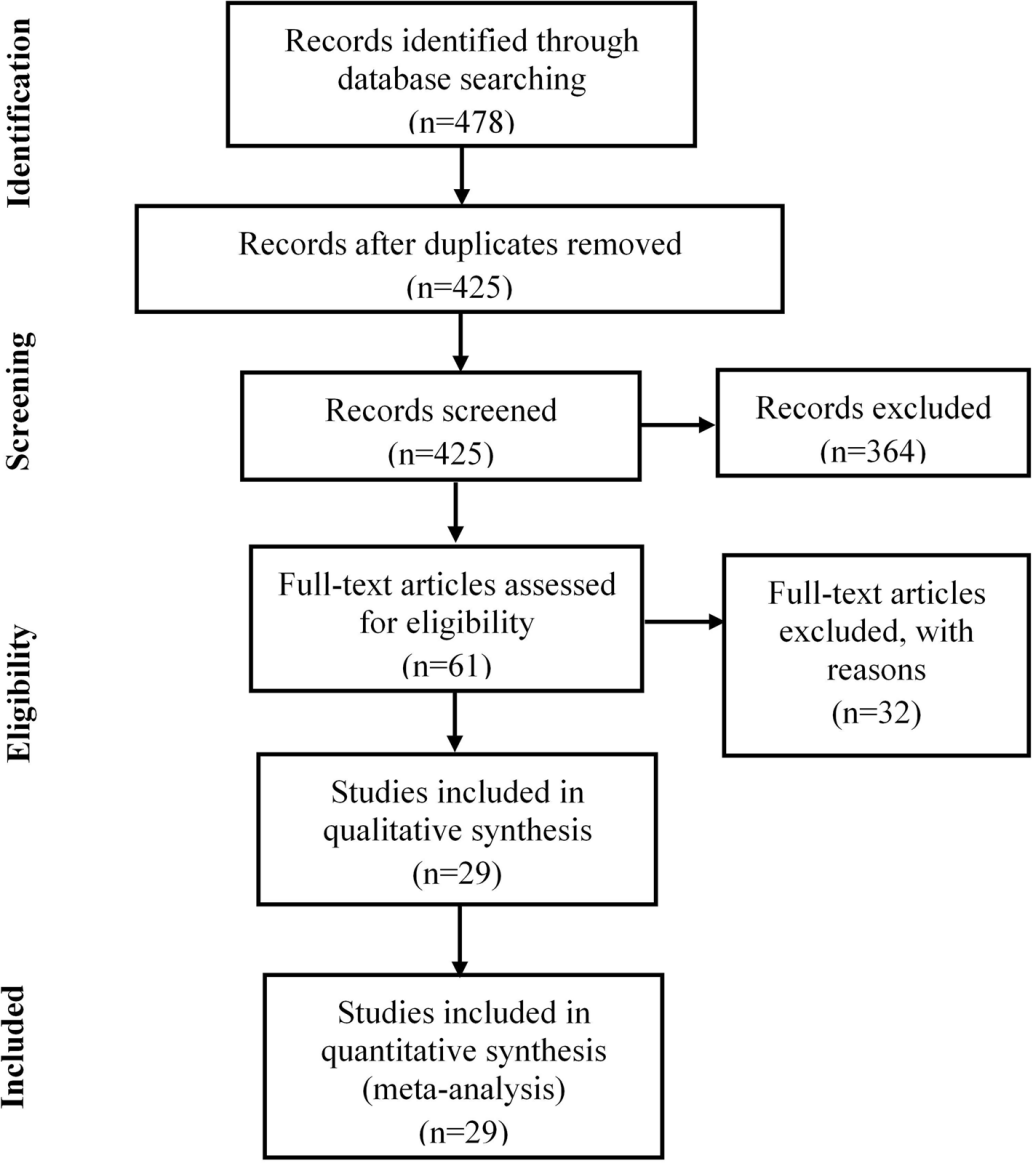
PRISMA flow diagram of the systematic literature search process

We initially identified 478 potentially eligible papers from the electronic databases. After excluding 53 duplicates, records (*n* = 425) were screened by reviewing titles and abstracts and excluded according to the exclusion criteria. Finally, the remaining 61 studies were further filtrated by reading the full text. As a result, 32 records were excluded, and 29 studies met the inclusion criteria. The included studies were divided into two groups (MSCs [31–38]; no MSCs [22, 24, 25, 39–56]) based on whether they applied the MSCs or not. The selection process is illustrated in Fig. 1. Screening and evaluation were conducted independently by two reviewers with resolution of disagreement by consensus or adjudication by a third reviewer.

### Quality assessment

The results of risk of bias for each study are shown in Tables 1 and 2. All studies had a score ≥ 5, which indicates a relatively high research quality. The subjects included were representative, and ascertainment of exposure was confirmed by secure record. Outcome assessment was based on medical records, and the follow-up period was sufficient for outcomes to occur.

**Table 1:** Characteristics of included studies in the meta-analysis (MSCs group)

Author, reference (publication year)	Study design	Recruitment period	Number of patients	Median age	Sex (M/F)	Type	Conditioning regimen	GVHD prophylaxis	Interval from diagnosis to treatment (M)	Neutrophil recovery (d)	Platelet engraftment	MSCs originals	NOS score
Wang Z-K [31] (2019)	Single-arm study	January 2014-December 2016	35	11.5 (3–18)	18/17	SAA/VSAA (19/16)	Busulfan (Bu) + cyclophosphamide (Cy) + antithymocyte globulin (ATG)	CsA + MMF + MTX	NA	14 (10–22)	18 (9–36)	BM	6
Yue C [32] (2018)	Cases	January 2014-January 2017	6	23 (15–31)	3/3	VSAA (6)	Busulfan (Bu) + cyclophosphamide (Cy) + antithymocyte globulin (rATG)	CsA + MMF + MTX	2 (1–3.5)	13 (9–19)	15.5 (10–23)	BM	5
Liu Z [33] (2017)	Single-arm study	March 2013-August 2015	44	24 (8–47)	29/15	SAA/VSAA (31/13)	Busulfan (Bu) + cyclophosphamide (Cy) + antithymocyte globulin (ATG)	CsA + MMF + MTX	31.2 (1–249)	12 (8–21)	19 (8–154)	BM	6
Xu L [34] (2018)	Single-arm study	June 2010-August 2013	24	16.5 (5–55)	14/10	SAA (24)	Cyclophosphamide (Cy) + antithymocyte globulin (rATG) + FLU or	CsA + ATG+ CD25Ab + mycophenolate material	NA	11 (10–25)	13 (10–25)	UC	6
Wu Y [35] (2017)	Single-arm study	January 2011-June 2016	77	9 (1–46)	39/38	SAAVSAA/SAA and PNH (72/5)	Cyclophosphamide (Cy) + antithymocyte globulin (ATG) + FLU ± busulfan	CsA + MMF + MTX + CD25Ab	7 (2–182)	12(8–21)	14 (9–30)	UC	5
Li XH [36] (2014)	Single-arm study	October 2006-October 2012	17	19 (4–29)	10/7	SAA/VSAA (8/9)	Cyclophosphamide (Cy) + antithymocyte globulin (ATG) + FLU	CsA + MMF + CD25Ab	3 (1–5)	12 (11–21)	14 (11–75)	UC	6
Wu Y [37] (2014)	Single-arm study	January 2007-June 2013	21	18 (4–31)	11/10	SAA/VSAA/SAA and PNH (7/12/2)	Cyclophosphamide (Cy) + antithymocyte globulin (rATG) + FLU or	CsA + MMF + CD25Ab + rATG	6 (1–128)	12 (8–21)	14(10–23)	UC	6
Wang Z [38] (2014)	Single-arm study	March 2010-April 2013	17	10 (4–19)	6/11	SAA/VSAA/2 HSCT (11/5/1)	BU + fludarabine + CY + ATG	CsA + MMF + MTX + CD25Ab	12 (1–44)	16 (9–25)	22 (9–95)	UC	6

*Abbreviations*: *SAA* severe aplastic anemia, *VSAA* very severe aplastic anemia, *ATG* antithymoglobulin, *CsA* cyclosporin A, *MSCs* mesenchymal stem cells, *haplo-HSCT* haplo-identical hematopoietic stem cell transplantation, *BU* busulfan, *Cy* cyclophosphamide, *MMF* mycophenolate mofetil, *FLU* fludarabine, *MTX* methotrexate, *GVHD* graft-versus-host disease

**Table 2:** Characteristics of included studies in the meta-analysis (no MSCs group)

Author (publication year)	Study design	Recruitment period	Number of patients	Median age	Sex (M/F)	Type	Conditioning regimen	GVHD prophylaxis	Interval from diagnosis to treatment (M)	Neutrophil recovery (d)	Platelet engraftment	MSCs originals	NOS score
Zhang YY [56] (2020)	Single-arm study	January 2013-Septemper 2018	35	43 (40–54)	23/11	SAA/VSAA (19/16)	BU + CY + ATG	CsA + MMF + MTX	NA	13 (9–21)	17 (10–102)	NA	6
Ma YR [55] (2020)	Single-arm study	NA	199	NA	106/93	NA	BU + CY + ATG	CsA + MMF + MTX	NA	NA	NA	NA	6
Liu LM [54] (2020)	Single-arm study	Septemper 2010-Septemper 2018	16	32 (8–55)	9/7	NA	ATG + rituximab	NA	NA	11 (9–20)	21 (13–112)	NA	6
Yang SW [22] (2019)	Single-arm study	NA	32	NA	21/11	NA	NA	NA	NA	NA	NA	NA	6
Xu LP5 [53] (2019)	Single-arm study	2006–2018	392	NA	223/167	NA	BU + CY + ATG	CsA + MMF + MTX	NA	12 (9–31)	14 (5–180)	NA	6
Hyery Kim [52] (2019)	Single-arm study	2008–2017	32	12.7 (1.4–21.7)	22/10	SAA/VSAA (20/12)	FLU, CY, ATG ± TBI	CsA + MMF	5.2 (1.2–106.8)	10 (9–30)	15.5 (13–60)	NA	5
Lu Y [51] (2018)	Single-arm study	Septemper 2012-Septemper 2016	41	13 (4–42)	25/16	SAA/VSAA(28/13)	FLU, CY, ATG	CsA + MMF + MTX	25 (6–45)	14 (10–21)	13 (3–56)	NA	6
Sung-Eun Lee [24] (2018)	Single-arm study	June 2012-December 2016	34	31.5 (17–59)	20/14	SAA/VSAA (11/23)	ATG + TBI + FLU	CsA + MTX	NA	12 (11–12)	14 (5–86)	NA	5
Cheng YF [50] (2018)	Single-arm study	December 2007-Septemper 2016	28	NA	NA	NA	NA	NA	2.75 (1–8)	12 (10–21)	NA	NA	6
Cao LQ [49] (2018)	Single-arm study	January 2006-December 2016	131	16 (2–45)	70/61	NA	NA	NA	NA	12 (10–31)	16 (7–276)	NA	5
Zhang P [48] (2017)	Single-arm study	June 2014-December 2015	8	14 (5–26)	5/3	NA	FLU, CY, ALG/TBI	CsA + MMF + MTX	NA	14.8 (11–20)	15.0 (11–21)	NA	6
Zhang Y [47] (2017)	Single-arm study	June 2010-December 2014	18	NA	NA	NA	NA	NA	NA	16 (12–26)	20 (17–35)	NA	5
Pei XY [46] (2017)	Single-arm study	January 2008-December 2015	81	14 (3–45)	50/31	SAA/VSAA (63/18)	NA	NA	NA	12 (10–22)	15 (7–150)	NA	6
Sarita Rani Jaiswal [45] (2017)	Single-arm study	January 2015-May 2016	20	NA	NA	NA	FLU, CY, ATG, melphalan	PTCy + sirolimus + CsA + MMF (abatacept)	NA	NA	NA	NA	5
Amy E. DeZern [44](2017)	Single-arm study	July 2011-August 2016	13	33 (11–69)	9/5	NA	FLU, CY, ATG ± TBI	PTCy+MMF + FK506	NA	19 (16–27)	28 (22–108)	NA	5
Zhu H [43] (2016)	Single-arm study	July 2002-November 2013	38	NA	12/24	SAA/VSAA (8/28)	FLU, CY, ATG ± TBI/BU	CsA + methotrexate (MTX) (*n* = 12) CsA + MTX + mycophenolate mofetil (MMF) (*n* = 22) MTX + tacrolimus (*n* = 4)	NA	NA	NA	NA	6
Liu L [42] (2016)	Single-arm study	July 2005-December 2013	26	26 (10–54)	15/11	SAA/VSAA/(16/6)	NA	NA	NA	12 (6–28)	19 (12–330)	NA	5
Ho Joon Im [41] (2015)	Single-arm study	NA	21	14 (3–21)	NA	NA	FLU, CY, ATG ± TBI	NA	NA	NA	NA	NA	6
Esteves I [25] (2015)	Single-arm study	July 2010-August 2014	16	17 (5–39)	11/5	NA	CY + FLU+ TBI, FLU + ATG (2)	MMF, CsA, Cy	NA	19 (16–29)	21 (20–29)	NA	6
Gao L [40] (2014)	Single-arm study	June 2007-December 2010	26	25.4 (18–41)	NA	SAA/VSAA (16/10)	FLU, CY, ATG	CsA + MMF + MTX + ATG	NA	13 (11–19)	13 (10–21)	NA	5
Jennifer Clay [39] (2014)	Single-arm study	NA	8	NA	NA	NA	CY + FLU + TBI	MMF + FLU + PTCY	NA	18.5 (16–23)	26 (21–27)	NA	5

*Abbreviations*: *SAA* severe aplastic anemia, *VSAA* very severe aplastic anemia, *ATG* antithymoglobulin, *CsA* cyclosporin A, *MSCs* bone marrow-mesenchymal stem cells, *haplo-HSCT* haplo-identical hematopoietic stem cell transplantation, *BU* busulfan, *Cy* cyclophosphamide, *MMF* mycophenolate mofetil, *FLU* fludarabine, *MTX* methotrexate, *GVHD* graft-versus-host disease, *TBI* total body irradiation

### Incidence of GVHD

**Fig. 2:**
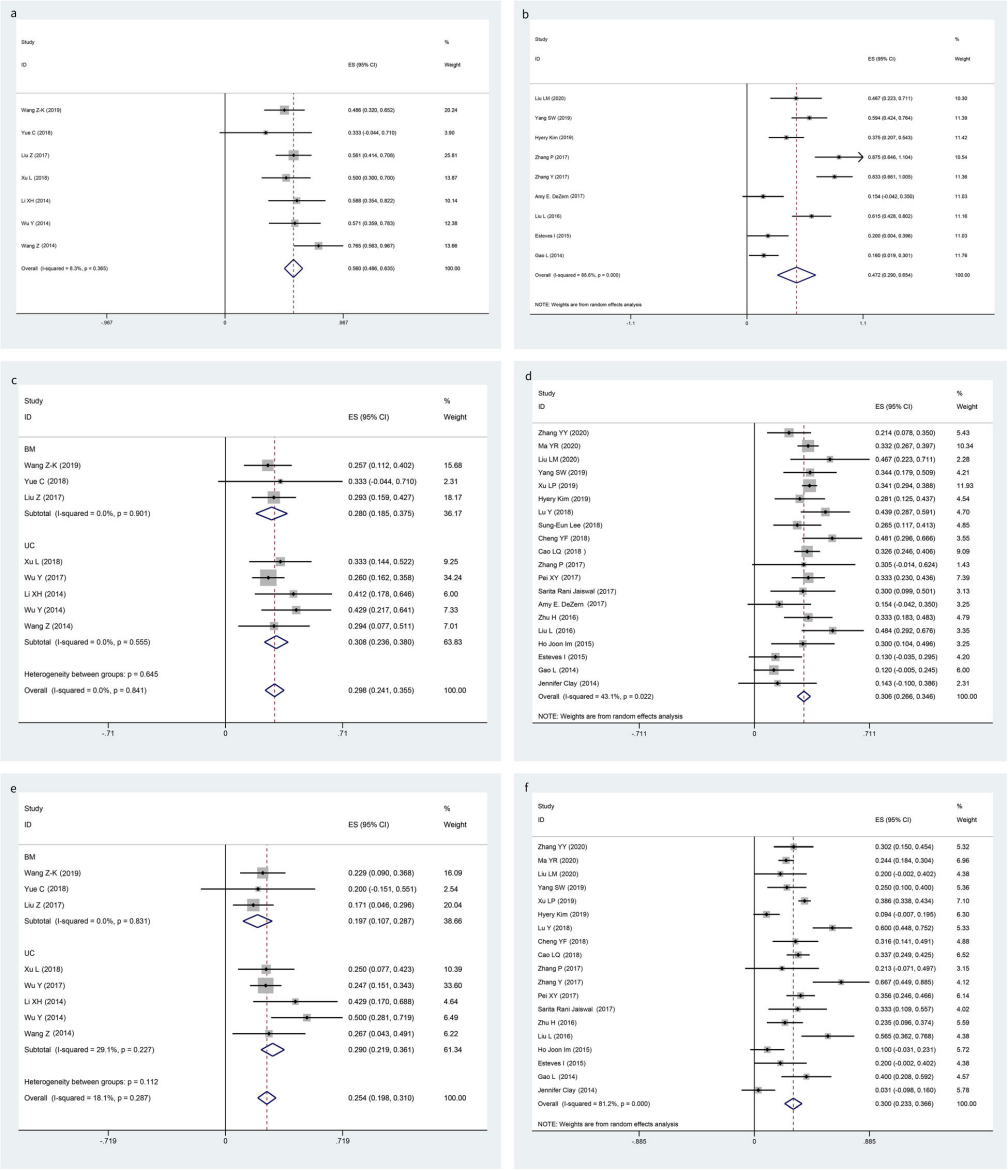
Forest plots for the pooled incidence of acute GVHD (**a** MSCs group; **b** no MSCs group), grade II–IV acute GVHD (**c** MSCs group; **d** no MSCs group), and chronic GVHD (**e** MSCs group; **f** no MSCs group)

The pooled results of aGVHD, grade II–IV aGVHD, and cGVHD in the MSCs group and no MSCs group are summarized in Table 3. Our meta-analysis revealed no significant heterogeneity in aGVHD (*I*-squared = 8.3%, *p* = 0.365), grade II–IV aGVHD (*I*-squared = 0.0%, *p* = 0.841), and cGVHD (*I*-squared = 18.1%, *p* = 0.287) in the MSCs group, so fixed models were applied. By pooling studies with no significant heterogeneity, we learned that the overall incidences of aGVHD (Fig. 2a), grade II–IV aGVHD (Fig. 2c), and cGVHD (Fig. 2e) were 56.0% (95% CI, 48.6 to 63.5%), 29.8% (95% CI, 24.1 to 35.5%), and 25.4% (95% CI, 19.8 to 31.0%) in the MSCs group respectively, while random models were applied in no MSCs group with heterogeneity in aGVHD (*I*-squared = 88.6%, *p* = 0.000), grade II–IV aGVHD (*I*-squared = 43.1%, *p* = 0.022), and cGVHD (*I*-squared = 81.2%, *p* < 0.001). The overall incidences of aGVHD (Fig. 2b), grade II–IV aGVHD (Fig. 2d), and cGVHD (Fig. 2f) were 47.2% (95% CI, 29.0 to 65.4%), 30.6% (95% CI, 26.6 to 34.6%), and 30.0% (95% CI, 23.3 to 36.6%) respectively. Results showed that there was insufficient evidence to detect a difference in the risk of aGVHD in the comparison of MSCs and no MSCs (OR 1.43, 95% CI 0.91 to 2.25; *p* = 0.123); the same situation was detected in grade II–IV aGVHD (OR 0.97, 95% CI 0.70 to 1.32; *p* = 0.889) and cGVHD (OR 0.79, 95% CI 0.56 to 1.11; *p* = 0.187) (Table 3). Subgroup analysis demonstrated no significant heterogeneity in subgroup between the use of UC-MSCs and BM-MSCs in the MSCs group (Fig. 2c, e).

**Table 3:** Pooled estimates in MSCs group and no MSCs group

Pooled estimates	Pooling model	Number of studies, haplo-HSCT + MSCs/haplo-HSCT alone	haplo-HSCT + MSCs (95% CI)	haplo-HSCT alone (95% CI)	OR (95% CI)	*p* value
aGVHD	Fixed/random	7/9	56.0% (48.6%, 63.5%)	47.2% (29.0%, 65.4%)	1.43 (0.91–2.25)	0.123
Grade II–IV aGVHD	Fixed/random	8/20	29.8% (24.1%, 35.5%)	30.6% (26.6%,34.6%)	0.97 (0.70–1.32)	0.889
cGVHD	Fixed/random	8/18	25.4% (19.8%, 31.0%)	30.0% (23.3%,36.6%)	0.79 (0.56–1.11)	0.187
2-year OS	Fixed/fixed	8/12	84.9% (80.4%, 89.3%)	85.2% (81.6%,88.8%)	0.98 (0.60–1.61)	1.000
Engraftment rate	Fixed/fixed	8/17	98.9% (96.4%, 100.0%)	98.6% (96.5%,99.8%)	1.02 (0.66–1.54)	1.000
CMV infection rate	Random/random	5/10	52.4% (31.6–73.1%)	64.1% (52.9–75.2%)	0.61 (0.40–1.92)	0.018

*Abbreviations*: *GVHD* graft-versus-host disease, *aGVHD* acute GVHD, *cGVHD* chronic GVHD, *MSCs* mesenchymal stromal cells, *haplo-HSCT* haplo-identical hematopoietic stem cell transplantation, *OR* odds ratio, *OS* overall survival, *CI* confidence interval, *CMV* cytomegalovirus

### Overall survival

**Fig. 3:**
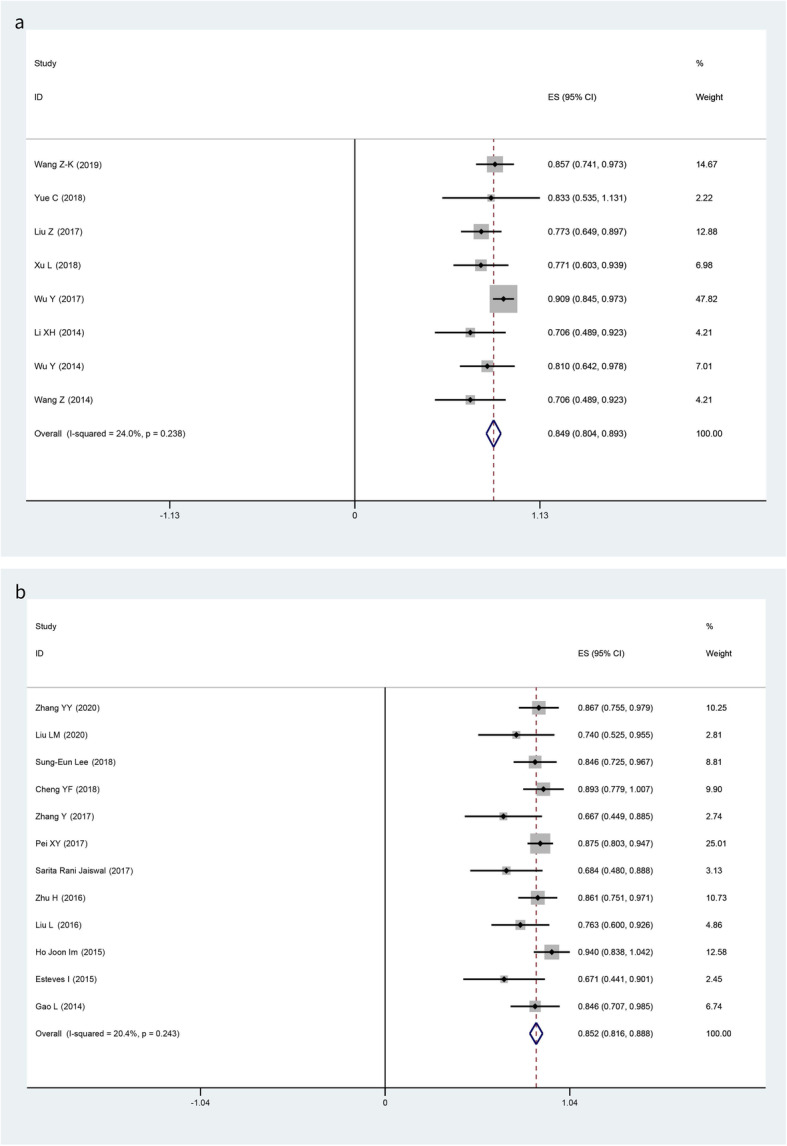
Forest plots for the pooled 2-year overall survival (**a** MSCs group; **b** no MSCs group)

Two-year OS were reported in 8 studies in the MSCs group and 12 studies in no MSCs group respectively without significant heterogeneity in both groups (MSCs: *I*-squared = 24.0%, *p* = 0.238; no MSCs: *I*-squared = 20.4%, *p* = 0.243) (Fig. 3a, b), so fixed models were applied. The pooled results of 2-year OS were 84.9% (95% CI, 80.4 to 89.3%) and 85.2% (95% CI, 81.6 to 88.8%) respectively (Fig. 3). There was no significant difference in 2-year OS in the comparison of MSCs and no MSCs (OR 0.98, 95% CI 0.60 to 1.61; *p* = 1.000) (Table 3).

### Engraftment rate and CMV infection rate

**Fig. 4:**
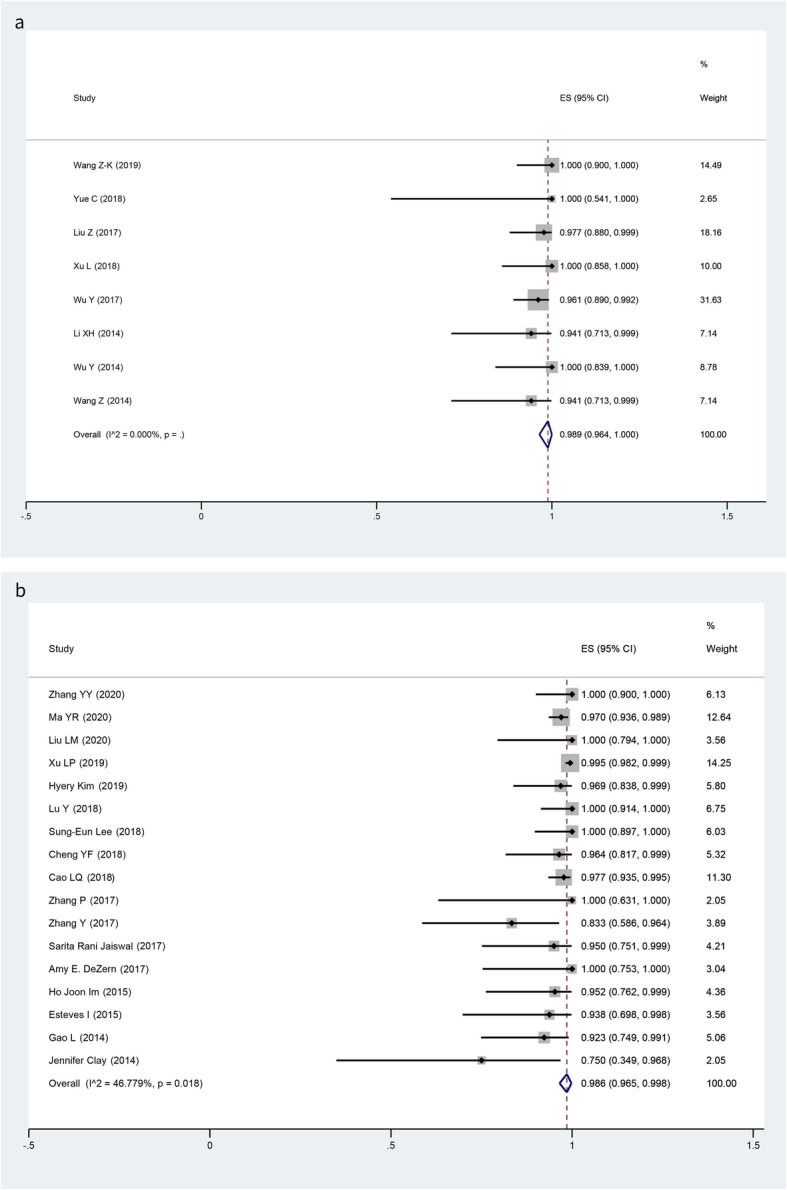
Forest plots for the pooled engraftment rate (**a** MSCs group; **b** no MSCs group)

**Fig. 5:**
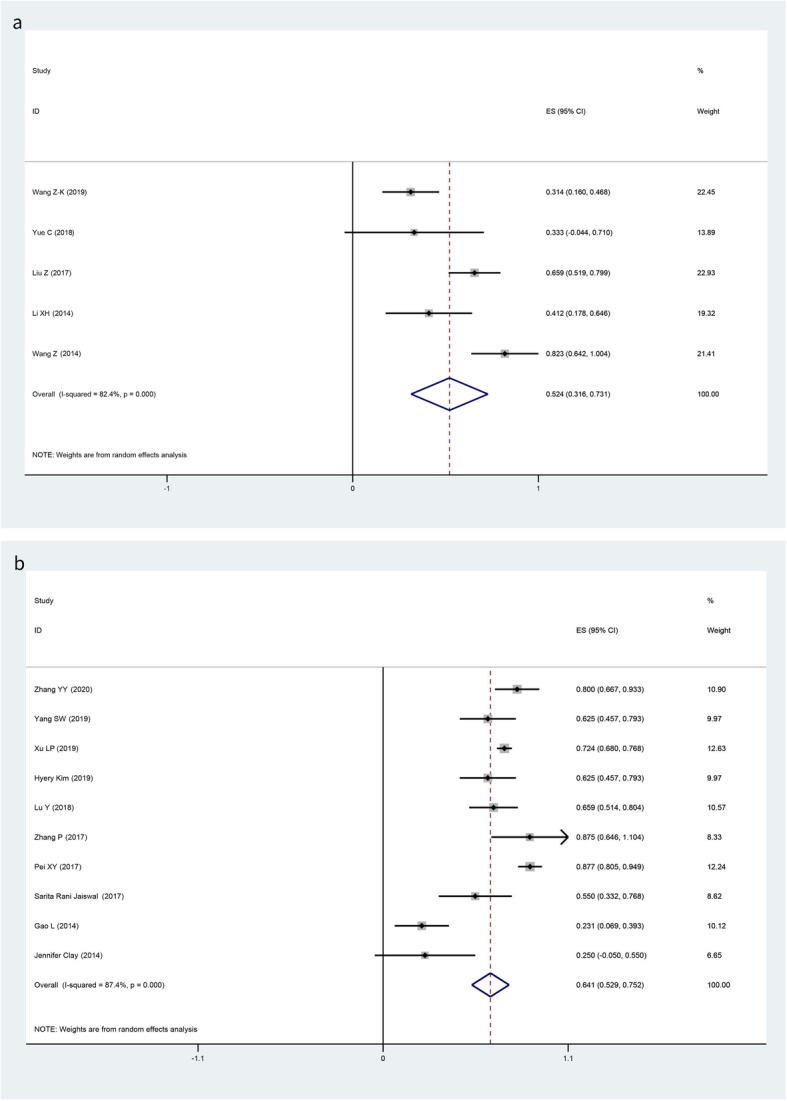
Forest plots for the pooled incidences of CMV viremia (**a** MSCs group; **b** no MSCs group)

Our meta-analysis revealed no significant heterogeneity in engraftment rate (MSCs: *I*-squared = 0%, *p* = 0; no MSCs: *I*-squared = 46.8%, *p* = 0.018). Four of eight studies achieved 100% hematopoietic reconstitution and full donor chimerism after haplo-HSCT with the administration of MSCs. The pooled results of engraftment rate were 98.9% (95% CI, 96.4 to 100.0%) and 98.6% (95% CI, 96.5 to 99.8%) respectively (Fig. 4). No significant difference was detected when compared MSCs with no MSCs (OR 1.02, 95% CI 0.66 to 1.54; *p* = 1.000). Random models were applied to pool the incidences of CMV infection because of significant heterogeneity detected in both groups, the pooled results were 52.4% (95% CI, 31.6 to 73.1%) and 64.1% (95%CI, 52.9 to 75.2%) respectively (Fig. 5). Likewise, no significant difference was observed (OR 0.61, 95% CI 0.40 to 1.92; *p* = 0.018) (Table 3).

## Discussion

This up-to-date meta-analysis comprehensively examined the published literature to evaluate the efficacy of co-transplantation of MSCs and haplo-HSCT in patients with SAA. To the best of our knowledge, this is the first meta-analysis to compare the clinical outcomes of MSCs with no MSCs in haplo-HSCT in patients with SAA. The results of our study are partially consistent with a previous meta-analysis examining the effects of MSCs post-transplantation of haplo-HSCT in hematological malignancies [15]. For example, we both concluded that MSCs make no difference in the incidences of aGVHD and CMV infection. They found a role of MSCs in reducing the incidences of cGVHD, while we did not.

Our study demonstrated no significant difference with regard to the pooled incidences of GVHD between MSCs and no MSCs groups. It is well known that GVHD remains the common and life-threatening complication limiting the widespread use of haplo-HSCT, as it associates with a high mortality and morbidity [57]. Since there were no controlled studies, we compared the incidence of aGVHD, grade II–IV aGVHD, and cGVHD in MSCs group and no MSCs group. Although the incidence of aGVHD was higher than no MSCs group, the incidences of grade II–IV aGVHD and cGVHD were lower than the pooled results in no MSCs group. However, no significant differences were found in pooled results between these two groups, which was different from the previous studies supporting a role of MSCs in reducing the incidence of GVHD [15, 27]. Despite these previous studies showing that MSCs are effective in GVHD prophylaxis or treatment, most of them were conducted in vitro or in hematological malignancies. Moreover, the conclusions were drawn in HSCT area without highlighting on haplo-HSCT [58–60]. Hence, MSCs may make little or no difference to the risk of GVHD compared to no MSCs in haplo-HSCT for SAA patients.

Among the MSCs group, four of eight studies achieved 100% hematopoietic reconstitution and full donor chimerism after the application of MSCs in haplo-HSCT, which is higher than no MSCs group (4/17). Although MSCs were higher than no MSCs group with regard to the pooled results of 2-year OS and engraftment rates in our report, no statistically significant differences were found. Furthermore, it is reported that infections are the other major causes of death after haplo-HSCT in addition to GVHD [61]. We calculated the rates of death due to infection. The pooled result was 9.5% (95% CI, 5.8 to 13.1%) in the included studies in the MSCs group, which was much lower than those reported by the Center for International Blood and Marrow Transplant Research (CIBMTR), for all haplo-HSCT transplants conducted between 2009 and 2010 (infection 13–18%) [62]. CMV infections are opportunistic infections caused by low immune function. A reduction in CMV infection after allo-HSCT can be achieved by hastening post-transplant immune reconstitution. Therefore, co-transplantation of MSCs and haplo-HSCT seemed like making no contribution to immune reconstitution in SAA patients.

It is reported that MSCs produce growth factors to aid tissue regeneration and accelerate the hematologic reconstitution [63]. The median post-HSCT times to neutrophil greater than 0.5 × 10^9^/L and platelet greater than 20 × 10^9^/L were summarized and listed in Table 1 and 2. The shortest and longest median time to achieve neutrophil engraftment and platelet engraftment were 11–14 days and 13–21 days respectively in the MSCs group and 10–19 days and 13–28 days respectively in the no MSCs group. Remarkably, all studies in the MSCs group reported results descriptively and stated that they observed either rapid engraftment [37] or a similar speed of engraftment after adding MSCs [31, 32], which may demonstrate a role for MSCs in the enhancement of engraftment in SAA patients who underwent haplo-HSCT.

According to the published papers, the treatment efficacy of MSCs varies among clinical trials, and MSC source might influence this [64]. The studies included in this meta-analysis used only BM-MSCs or UC-MSCs. Therefore, we conducted a subgroup meta-analysis for GVHD prevention according to MSC source. Consequently, the incidence of GVHD shows no significant difference with regard to the use of UC-MSCs versus BM-MSCs. Besides, we conducted “influence analysis” in Stata to explore the source of heterogeneity in no MSCs group. We could reasonably infer that studies (Jennifer et al. [39] and Gao et al. [40]) were one of the most important sources resulting in the heterogeneity of aGVHD and cGVHD respectively. Both of them were conducted in earlier years with different conditioning regimes and prophylaxis measures.

There are some limitations in our meta-analysis. First, there may be a risk of confounding biases because various baseline characteristics or co-interventions including age, gender, donor type, conditioning regimen, and MSC originals may affect the treatment outcomes in SAA patients after haplo-HSCT; they were not fully controlled in this study. In addition, patients in no MSCs group usually had high heterogeneity. Although we tried to decrease the bias through statistical methods, sometimes errors were unavoidable. Second, because SAA is a rare disorder, few prospective control trials between the MSCs and no MSCs group are available so far, and all the included studies had small sample sizes. Besides, all were single-arm studies and case series that lacked a control group and likely suffered from a high risk of selection bias. Last but not least, we could not assess publication bias using funnel plots because we only had single-arm studies and case series.

In conclusion, our meta-analysis indicates that the prophylactic use of MSC co-transplantation does not reduce the incidence of GVHD and improve 2-year OS in patients with SAA undergoing haplo-HSCT. Hence, the general co-transplantation of MSCs in routine clinical practice for SAA haplo-HSCT recipients is not recommended. However, since there is no direct evidence from comparative study to support this conclusion, more prospective, randomized controlled trials (RCTs) are needed to confirm whether MSCs convey a definite benefit for haplo-HSCT for SAA patients.

## Data Availability

All supporting data are included in the article and Additional file.
